# Editorial: Strategies for the Discovery of Fungal Natural Products

**DOI:** 10.3389/fmicb.2022.897756

**Published:** 2022-05-05

**Authors:** Fernando Reyes, Gerald F. Bills, Rosa Durán-Patrón

**Affiliations:** ^1^Centro de Excelencia en Investigación de Medicamentos Innovadores en Andalucía, Fundación MEDINA, Granada, Spain; ^2^Department of Plant Biology, Rutgers University, New Brunswick, NJ, United States; ^3^Departamento de Química Orgánica, Universidad de Cádiz, Cádiz, Spain

**Keywords:** fungus, cryptic metabolite, OSMAC, epigenetic, co-cultivation, fungal cultivability, accessory chromosomes

Fungal natural products are characterized by a wide spectrum of biological activities that ensure the adaptation of fungi to their environment and that mediate their interactions with other organisms. Some of these natural products are promising sources of new lead compounds, especially for the pharmaceutical, cosmetic, agrochemical and food industries. However, others known as mycotoxins pose risks to human and animal health or are involved in plant diseases as pathogenicity factors. Therefore, the discovery of new fungal natural products is critical to assessing both the utility and risks of these compounds (Hautbergue et al., 2018).

Technological advances in genome sequencing and bioinformatics have substantially increased the number of sequenced microbial genomes. This advance in genomic information has revealed an inconsistency between the number of genes clusters encoding the production of secondary metabolites and the actual number of natural products isolated from a given microorganism. Many of these gene clusters are generally considered silent, as they are not expressed under laboratory conditions (Romano et al., 2018).

Recently, new genetic and cultivation-based strategies have been developed aimed at awakening these silent gene clusters. Methods that use genetic engineering techniques require a relatively sophisticated knowledge of the biology of the producing or surrogate host organisms. In contrast, global physiological alterations can be triggered by approaches based on the modification of the growth conditions without genetically manipulating the organism. This historically important and empirical approach, commonly called OSMAC (one strain—many compounds), exploits the fact that changes in the cultivation parameters, such as nutrients, temperature, salinity, aeration, or even the flask shape, can elicit production and discovery of new natural products (Bode et al., 2002; Bills et al., 2012; Tormo et al., 2012; Pinedo-Rivilla et al., 2022). In their article published in this Research Topic, Gao et al. used this approach to isolate 11 new lactam derivatives, aplosporelins A-K, structurally related to the known co-occurring pramanicin A, from the endophytic fungus *Aplosporella javeedii*. All these compounds were detected when the fungus was grown on rice medium supplemented with 3.5% NaNO_3_ or 3.5% monosodium glutamate but were absent in untreated rice medium. Pramanicin A showed strong cytotoxicity against human lymphoma (Ramos) and leukemia (Jurkat J16) cells with IC_50_ values of 4.7 and 4.4 μM, respectively, proving that this approach is an interesting alternative for the discovery of novel cryptic natural products of pharmacological interest.

The directed biosynthesis approach allows for the manipulation and design of new natural products in wild type filamentous fungi and pathway-blocked mutants through the addition of alternative or non-natural biosynthetic precursors analogs of the target pathway. In this issue, Subko et al. obtained 21 novel asperphenamate analogs, which were characterized by HRMS/MS, after growing *Penicillium astrolabium* IBT 28865 on media enriched with proteogenic and non-proteogenic amino acids. Asperphenamates are peptides with antitumour activity, so this study was conducted to produce derivatives that provide a solution to the increasing resistance toward anticancer drugs. Asperphenamate Y was active against the breast adenocarcinoma cell line MCF7, suggesting that the presence of tyrosine at the non-reduced amino acid moiety may be essential for the observed activity. In addition, asperphenidine F1 was active against the pancreas carcinoma cell line MiaPaca, suggesting that nicotinic acid analogs are more active than the benzoic acid analogs.

As an alternative approach, the addition of chemical elicitors or epigenetic modifiers has been used to empirically activate silent genes. Elicitors are signal compounds or general physiological effectors that stimulate the activation or synthesis of other compounds. For example, metals have been used to chemically induce the production of new cryptic sesquiterpenoids in *Botrytis cinerea*. Cultures of this fungus on a malt-agar medium supplemented with different sublethal amounts of copper sulfate revealed new sporogenic (+)-4-epieremophil-9-en-11-ols (Pinedo et al., 2016). DNA methylation and modulation of chromatin structure have important effects on global gene expression, so the use of epigenetic modifiers can induce transcription of silent gene clusters. 5-Aza-2-deoxycytidine, a DNA methyltransferase inhibitor, was used to induce the biosynthesis of three previously undescribed polyketides with a pyran-2-one skeleton from *Penicillium herquei* (Guo et al., 2020).

Biosynthesis of cryptic fungal natural products can also be elicited by a biotic approach. The most commonly used technique is co-culture with other bacterial and/or fungal strains. Li et al. (2014) isolated a new cyclic tetrapeptide, cyclo-(l-leucyl-*trans*-4-hydroxy-l-prolyl-d-leucyl-*trans*-4-hydroxy-l-proline), from the co-culture broth of two mangrove fungi, *Phomopsis* sp. K38 and *Alternaria* sp. E33.

In addition to the above-mentioned activation of the silent gene clusters of cultivated strains, an obvious way to expand chemodiversity would be to increase the diversity of species in culture collections by improving the cultivability of the fungi. In this Research Topic, Rämä and Quandt reviewed in depth the techniques developed to increase the cultivability of filamentous fungi, which include culture media formulations and the use of known chemical growth factors, *in situ* cultivation and current synthetic biology approaches based on the knowledge of sequenced genomes.

The fungal natural product community is focused on unlocking the cryptic metabolome and linking secondary metabolites with their respective biosynthetic gene clusters. Fungal secondary metabolism can vary within populations of some plant pathogen species due to the presence of lineage-specific accessory chromosomes (ACs) within the genome of strains. ACs are strain- or pathotype-specific chromosomes that exist alongside the core chromosomes of a species. Also known as “B-type,” “supernumerary,” or “lineage-specific” chromosomes, ACs are generally not considered essential for the survival of the organism. Among pathogenic fungal species, these chromosomes harbor pathogenicity or virulence factor genes, several of which are known to encode for secondary metabolites that are involved in plant tissue invasion.

In this special issue, Witte et al. reviewed the secondary metabolism associated with ACs in filamentous fungi and the role these chromosomes play in the evolution of secondary metabolite gene clusters. Expression of AC-associated gene clusters expands both the diversity and quantity of mycotoxins and other virulence factors produced, allowing isolates to invade and adapt to specific plant hosts. Untargeted metabolomics is a potent emerging tool for exploring the evolving chemical space associated with these adaptations. Such biosynthetic gene clusters on ACs may also provide insights into the evolution of fungal natural products, as the dynamic nature of ACs may promote diversification of natural product families.

In summary, this Research Topic provides readers an overview of some of the latest strategies for the discovery and characterization of new fungal natural products. The traditional methods of fungal cultivation along with a focus on the easiest to culture species has constrained the perceived chemical landscape of fungal fermentations to a limited number of metabolic pathways. In turn, these constraints have led to a stagnation in the rate of discovery of new natural products and increased rediscovery of known compounds. We therefore hope that this Research Topic can increase awareness and open alternative avenues for the discovery of new bioactive compounds.

## Author Contributions

RD-P wrote the first draft of the Editorial. All authors made a substantial, direct, and intellectual contribution to the work and approved the submitted version.

## Conflict of Interest

The authors declare that the research was conducted in the absence of any commercial or financial relationships that could be construed as a potential conflict of interest.

## Publisher's Note

All claims expressed in this article are solely those of the authors and do not necessarily represent those of their affiliated organizations, or those of the publisher, the editors and the reviewers. Any product that may be evaluated in this article, or claim that may be made by its manufacturer, is not guaranteed or endorsed by the publisher.
